# Outcome of Stroke Patients with Unknown Onset and Unknown Time Last Known Well Undergoing Endovascular Therapy

**DOI:** 10.1007/s00062-022-01188-5

**Published:** 2022-07-07

**Authors:** Sebastian Stösser, Felix J. Bode, Julius N. Meissner, Johannes M. Weller, Christine Kindler, Malte Sauer, Daniel Paech, Christoph Riegler, Christian H. Nolte, Amitis Pourian, Joachim Röther, Nadja Selo, Ulrike Ernemann, Sven Poli, Rosa Marie Eckert, Georg Bohner, Korbinian Scherling, Franziska Dorn, Gabor C. Petzold, GSR-ET study group

**Affiliations:** 1 Division of Vascular Neurology, Department of Neurology, University Hospital Bonn, Venusberg-Campus 1, 53127 Bonn, Germany; 2 Department of Diagnostic and Interventional Neuroradiology, University Hospital Bonn, Bonn, Germany; 3 Klinik und Hochschulambulanz für Neurologie, Charité—Universitätsmedizin Berlin, corporate member of Freie Universität Berlin and Humboldt Universität zu Berlin, Berlin, Germany; 4 Department of Neurology, Asklepios Hospital Hamburg Altona, Semmelweis University Campus Hamburg, Hamburg, Germany; 5 Department of Neuroradiology, University Hospital Tübingen, Tübingen, Germany; 6 Department of Neurology, University Hospital Tübingen, Tübingen, Germany; 7 Department of Neurology, Charité-Universitätsmedizin Berlin, Berlin, Germany; 8 Department of Neuroradiology, Charité—Universitätsmedizin Berlin, Berlin, Germany; 9 Department of Neuroradiology, Ludwig Maximilians University Munich, Campus Grosshadern, Munich, Germany; 10 Center for Stroke Research Berlin (CSB), Charité—Universitätsmedizin Berlin, Berlin, Germany; 11 Berlin Institute of Health at Charité—Universitätsmedizin Berlin, Berlin, Germany

**Keywords:** Acute ischemic stroke, Large vessel occlusion stroke, Stroke registry, Thrombectomy, Modified Rankin Scale

## Abstract

**Purpose:**

Endovascular treatment (ET) in patients with large vessel occlusion stroke (LVOS) with unknown onset or an extended time window can be safe and effective if patients are selected by defined clinical and imaging criteria; however, it is unclear if these criteria should also be applied to patients with unknown onset and unknown time last known well. In this study, we aimed to assess whether absent information on the time patients were last known to be well impacts outcome in patients with unknown onset LVOS.

**Methods:**

We analyzed patients who were enrolled in the German Stroke Registry-Endovascular Treatment between 2015 and 2019. Patients with unknown onset and unknown time last known well (LKWu) were compared to patients with known onset (KO) and to patients with unknown onset but known time last known well (LKWk) regarding clinical and imaging baseline characteristics and outcome.

**Results:**

Out of 5909 patients, 561 presented with LKWu (9.5%), 1849 with LKWk (31.3%) and 3499 with KO (59.2%). At 90 days, functional independency was less frequent in LKWu (27.0%) compared to KO (42.6%) and LKWk patients (31.8%). These differences were not significant after adjusting for confounders. A main confounder was the initial Alberta stroke program early CT score.

**Conclusion:**

The LKWu patients had a similar outcome after ET as KO and LKWk patients after adjusting for confounders. Thus, ET should not be withheld if the time last known well is unknown. Instead, LKWu patients may be selected for ET using the same criteria as in LKWk patients.

## Introduction

Endovascular treatment (ET) in patients with acute large vessel occlusion stroke (LVOS) with unknown time of symptom onset or an extended time window can be safe and effective if defined criteria are met. The DAWN (DWI or CTP Assessment with Clinical Mismatch in the Triage of Wake-Up and Late Presenting Strokes Undergoing Neurointervention with Trevo) trial showed that ET improved clinical outcome in patients who were last known to be well 6–24 h before ET and had a mismatch between clinical severity and infarct volume [1]. The DEFUSE 3 (Endovascular Therapy Following Imaging Evaluation for Ischemic Stroke 3) trial showed a benefit for ET in patients who were last known to be well 6–16 h before ET and were selected by perfusion imaging [2]; however, in both trials patients in whom the time last known well was unknown were excluded. It is therefore unclear if patients with an unknown onset and unknown time last known well (LKWu) differ from patients with a known onset (KO) and patients with an unknown onset but known time last known well (LKWk) in clinical and imaging characteristics and in clinical outcome. If LKWu patients had a similar outcome to patients with a known time window after ET, these patients may be selected for ET by imaging criteria and may not be excluded from treatment based on the unclear time window.

In this study we compared LKWu patients to KO and LKWk patients regarding clinical and imaging baseline characteristics and functional outcome in a large multicenter cohort representing acute stroke care in both university and community hospitals.

## Methods

The data analyzed in this study were derived from patients included in the German Stroke Registry-Endovascular Treatment (GSR-ET) between June 2015 and December 2019. The GSR-ET is an ongoing, open-label, prospective, multicenter registry of 25 university and community hospitals in Germany enrolling consecutive patients with LVOS undergoing ET. The inclusion criteria are a clinical diagnosis of acute ischemic stroke, intention to perform ET and age ≥ 18 years. The decision on which imaging modalities are used and which patient is selected for ET is made by the local investigators in each hospital based on national guidelines. There are no exclusion criteria. A detailed description of the GSR-ET study design and a report of the main outcome of the patients included between June 2015 and April 2018 study were previously published [3, 4]. The time of symptom onset and, in cases of unknown onset, the time of last known well were collected prospectively in all patients. To ensure the valid documentation of these items, cases with missing data for any of the following items, which were considered important for clinical outcome, were excluded from the analysis: age, sex, premorbid modified Rankin Scale (mRS), National Institutes of Health Stroke Scale (NIHSS) scores at admission, intravenous thrombolysis, and successful recanalization. Additionally, missing data on the onset of symptoms were analyzed for each center to exclude a center-specific bias due to systematically missing data.

Symptomatic intracranial hemorrhage was evaluated retrospectively according to the European Cooperative Acute Stroke Study (ECASS) II definition [5]. Imaging studies of LKWu and LKWK patients were retrospectively evaluated in five participating centers for the following criteria: mismatch between MRI diffusion weighted and fluid attenuated inversion recovery imaging (DWI-FLAIR mismatch), a qualitative mismatch between infarct core and penumbra (qualitative perfusion mismatch), and fulfilment of DAWN and DEFUSE 3 inclusion criteria [1, 2, 6]. Imaging data of a previous analysis of the GSR-ET database were included as well [7].

Statistical analyses were performed using the Statistical Package for Social Sciences version 25.0.0.0 (IBM SPSS Statistics, Armonk, NY, USA). Differences in baseline data were assessed using Kruskal-Wallis tests for ordinal or metric data and Pearson χ^2^-tests for nominal data with Bonferroni correction for multiple comparisons. Differences in outcome data were assessed using multivariable logistic, ordinal and linear regression models where appropriate. All tests were 2‑tailed. Statistical significance was determined at an α level of 0.05. A Bonferroni correction for multiple comparisons was applied to *p* values.

## Results

The data of 6635 patients enrolled into the GSR-ET between June 2015 and December 2019 were available. Due to missing data for relevant key items, 563 patients were excluded from the analysis. Furthermore, the patients of one center (*n* = 163) were excluded from the analysis because of systematically missing data on the time of stroke onset. Among the remaining 5909 patients, 3499 (59.2%) patients presented with a known onset of symptoms (KO), 1849 (31.3%) patients presented with an unknown onset of symptoms, but a known time last known well (LKWk) and 561 (9.5%) patients presented with an unknown onset of symptoms and an unknown time last known well (LKWu).

Table 1 shows how LKWu patients compared to KO and LKWk patients regarding demography, medical history and stroke assessment and treatment. Notably, LKWu patients had significantly lower Alberta stroke program early CT (ASPECT) scores, a higher rate of MRI imaging, a lower rate of intravenous thrombolysis, a longer time from admission to flow restoration, and a lower rate of successful recanalization compared to KO patients. Compared to LKWk patients, LKWu patients had a significantly lower rate of intravenous thrombolysis, and a longer time from admission to flow restoration. Advanced imaging criteria indicating a mismatch between infarct core and salvageable brain tissue (qualitative perfusion mismatch, DAWN and DEFUSE 3 criteria) showed no significant differences between LKWk and LKWu patients. A DWI-FLAIR mismatch was less often observed in LKWu compared to LKWk patients, but this difference was not significant.

**Table 1:** Demography, medical history and stroke assessment and treatment of patients with unknown onset and unknown time last known well (LKWu) undergoing endovascular therapy compared to patients with known onset (KO) and to patients with unknown onset but known last known well (LKWk)

Variables	LKWu	KO	LKWk
Age, years, median (Q1–Q3)	76 (65–83)	75 (64–82)	77 (66–83)
Sex, female, *n* (%)	300 (53.5)	1701 (48.6)	982 (53.1)
Arterial hypertension, *n* (%)	421 (75.7)	2674 (76.8)	1458 (79.1)
Diabetes mellitus, *n* (%)	133 (23.9)	751 (21.6)	397 (21.6)
Dyslipidemia, *n* (%)	210 (37.8)	1371 (39.5)	750 (40.8)
Atrial fibrillation, *n* (%)	239 (43.0)	1452 (41.7)	770 (41.9)
Smoking, *n* (%)	132 (25.6)	805 (25.3)	450 (26.8)
Premorbid mRS, median (Q1–Q3)	0 (0–1)	0 (0–1)^a^	0 (0–1)
NIHSS admission, median (Q1–Q3)	15 (9–19)	14 (9–19)	15 (10–19)
Time onset-admission, min, median (Q1–Q3)	–	125 (60–208)	–
Time LKW-admission, min, median (Q1–Q3)	–	–	402 (220–715)
ASPECTS, median (Q1–Q3)	8 (7–10)	9 (8–10)^a^	8 (7–10)
Perfusion imaging done, *n* (%)	287 (55.5)	1645 (50.6)	995 (57.6)
Qualitative perfusion mismatch, *n* (%)	28 (90.3)	–	131 (96.3)
DEFUSE 3 criteria fulfilled, *n* (%)	14 (66.7)	–	76 (78.4)
DAWN criteria fulfilled, *n* (%)	12 (48.0)	–	62 (50.0)
MRI imaging done, *n* (%)	87 (15.5)	215 (6.1)^a^	211 (11.4)
DWI-FLAIR mismatch present, *n* (%)	8 (47.1)	–	35 (70.0)
Intravenous thrombolysis, *n* (%)	145 (25.9)	2192 (62.9)^a^	650 (35.2)^a^
Time admission-flow restoration, min, median (Q1–Q3)	135 (97–179)	110 (80–148)^a^	118 (88–160)^a^
Successful recanalization (final mTICI 2b/3), *n* (%)	448 (79.9)	3007 (85.9)^a^	1546 (83.6)

*LKWu* time last known well unknown, *KO* known onset, *LWKk* time last known well known, *mRS* modified Rankin scale, *NIHSS* National Institutes of Health Stroke Scale, *ASPECTS* Alberta Stroke Programme Early Computed Tomography Score, *MRI* magnetic resonance imaging, *DWI-FLAIR* diffusion weighted imaging-fluid attenuated inversion recovery, *mTICI* modified thrombolysis in cerebral infarction scale

^a^Indicates a significant difference compared to LKWu patients (*p* < 0.05)

Table 2 shows the analysis of clinical and radiological outcome measures using adjusted multivariable analyses. The following confounders were included in the analyses: age, sex, premorbid modified Rankin Scale (mRS), National Institutes of Health Stroke Scale (NIHSS) scores at admission, ASPECT score, intravenous thrombolysis, time from admission to flow restoration and successful recanalization. The LKWu patients had significantly higher NIHSS scores at 24 h compared to KO and LKWk patients. At discharge, the NIHSS scores of LKWu patients were significantly higher than in KO, but not compared to LKWk patients. The length of hospitalization was significantly longer in LKWu patients than in KO, but not compared to LKWk patients.

**Table 2:** Clinical and radiological outcome of patients with unknown onset and unknown time last known well (LKWu) undergoing endovascular therapy compared to patients with known onset (KO) and to patients with unknown onset but known last known well (LKWk)

	LKWu	KO	LKWk
*24* *h follow-up*			
NIHSS, median (Q1–Q3)	14 (6–21)	9 (3–18)^a^	12 (5–20)^a^
Any intracranial hemorrhage, *n* (%)	67 (11.9)	389 (11.1)	223 (12.1)
Symptomatic intracranial hemorrhage, *n* (%)	29 (5.2)	116 (3.3)	67 (3.6)
*Discharge follow-up*			
NIHSS, median (Q1–Q3)	8 (2–16)	4 (1–12)^a^	7 (3–15)
Length of stay, days, median (Q1–Q3)	10 (6–15)	8 (5–13)^a^	9 (6–14)
Death, *n* (%)	103 (18.8)	517 (15.0)	319 (17.5)
*90 days follow-up*			
Functional independency (mRS ≤ 2), *n* (%)	126 (27.0)	1297 (42.6)	514 (31.8)
mRS, median (Q1–Q3)	4 (2–6)	3 (1–6)^a^	4 (2–6)
Death, *n* (%)	149 (32.0)	777 (25.5)	524 (32.4)

*LKWu* time last known well unknown, *KO* known onset, *LWKk* time last known well known, *NIHSS* National Institutes of Health Stroke Scale, *mRS* modified Rankin scale

^a^Indicates a significant difference compared to LKWu patients (*p* < 0.05 in multivariable regression adjusted for age, sex, premorbid mRS, NIHSS at admission, initial Alberta Stroke Programme Early Computed Tomography Score, intravenous thrombolysis, time admission-flow restoration, successful recanalization)

The clinical outcome of LKWu patients at 90 days, indicated by the frequency of patients with functional independency (mRS ≤ 2), and by the rate of death, was numerically worse compared to KO patients, but these differences were not significant in multivariable analyses (adjusted odds ratio, aOR, 95% confidence interval, CI, for functional independency of LKWu patients compared to KO patients: 0.76, 0.53–1.09; aOR for death: 0.97, 0.68–1.38). The median mRS at 90 days was significantly higher in LKWu patients compared to KO patients (aOR for a higher median mRS: 1.45, 1.15–1.82). When the ASPECT score was removed from the regression model, the aOR for functional independency became statistically significant (0.61, 0.44–0.83). The same held true for the time from admission to flow restoration (aOR: 0.62, 0.46–0.84). The removal of other single predictors did not lead to similar changes. Removing single predictors did not change the aOR for a higher median mRS and for death at 90 days. The clinical outcome at 90 days of LKWu patients did not differ significantly from LKWk patients.

## Discussion

In this multicenter cohort of 5909 LVOS patients undergoing endovascular therapy, 9.5% of all patients presented with an unknown time of symptom onset and an unknown time last known well. These LKWu patients differed from KO and LKWk patients in some aspects of acute diagnostic and therapeutic management, such as more extensive early signs of ischemia, a higher rate of MRI imaging, a lower rate of intravenous thrombolysis, a lower rate of successful recanalization, and longer in-hospital workflow times. These differences were expected based on the unclear, potentially longer time window and the subsequently more thorough work-up before ET. Notably, the rate of intravenous thrombolysis was surprisingly high in LKWu patients (25.9%). Without the evidence of a DWI-FLAIR mismatch on MRI, intravenous thrombolysis is not recommended in LKWu patients. As only 15.5% of LKWu patients were imaged by MRI, the majority of LKWu patients received off-label intravenous thrombolysis. Possible reasons why local investigators may have decided for this include evidence of salvageable brain tissue on perfusion imaging, a high ASPECT score, or an imminent long transport in a drip-and-ship setting. The lower rate of successful recanalization in LKWu patients compared to KO patients was expected based on previous work demonstrating a decreasing rate of successful recanalization over time [8]. This may be related to changes of thrombus composition and thrombus elongation over time [9, 10].

The LKWu patients had a worse clinical outcome than KO patients at 24 h and hospital discharge. At 90 days, the outcome of LKWu patients was numerically worse than in KO patients, but this difference was not significant after adjusting for confounders except for the mRS shift. Further analysis revealed that the lower ASPECT score in LKWu patients was a main confounder. This suggests that the ASPECT score at admission, indicating more extensive early signs of ischemia, is a more important predictor of clinical outcome after LVOS than the time of onset to treatment. Compared to LKWk patients, LKWu patients showed a worse 24 h outcome, but no significant differences at later time points. Advanced imaging studies showed that LKWu patients fulfilled the DAWN and DEFUSE 3 criteria as frequently as LKWk patients, indicating a similar rate of mismatch between a relatively small infarct core and salvageable brain tissue in both groups, which may explain the similar outcome of both groups.

Previous studies did not report specifically on the outcome of LKWu patients undergoing ET, but LKWu patients were included in some studies on patients with unknown onset stroke. Similar to our data, Escalard et al. did not report significantly different outcomes after ET in patients with unknown onset of stroke compared to controls [11]; however, patients with unknown onset of stroke were selected for ET by an MRI indicating stroke onset within 6 h before admission in that study, and the time last known well was not reported. Thus, the comparability of these results to our data is limited. In contrast, Bücke et al. showed that patients with daytime unwitnessed stroke, which included an unspecified proportion of LKWu patients, have a worse outcome 3 months after ET than patients with a known onset [12]; however, the multivariable analyses were not adjusted for the ASPECT score that we identified as key predictor, which may explain the difference to our results. Tortuyaux et al. observed a comparable outcome of patients with unknown onset at 3 months, including 34% of LKWu patients, compared to controls with known onset after adjustment for confounders [13]; however, only a subset of patients in that study had LVOS and underwent ET, limiting comparability to our data.

The ASPECT score being a main predictor of clinical outcome in LKWu patients raises the question if these patients might be selected for ET based on the ASPECT score alone without advanced imaging. While this question cannot be addressed directly using our data, previous studies demonstrated that the treatment effect of ET in patients selected by ASPECT score diminishes over time at 6 h and later after stroke onset, arguing against employing this strategy in patients with an unknown onset [14, 15].

This study has several limitations. Patients might have been falsely classified as LKWu if information on the onset of symptoms were available to the clinicians at admission, but these data were not included in the registry database. Several measures were taken to minimize this risk, but it still represents a potential source of selection bias. Furthermore, the decision for ET was at the discretion of local investigators without registry-wide criteria, such as a fixed ASPECT score cut-off. Moreover, it is unknown how many patients were excluded from ET by local investigators since those patients were not included in the registry. This may have caused heterogeneity of the LKWu group, limiting the generalizability of our conclusions.

To our knowledge, this is the first study comparing the clinical outcome of LKWu to KO and LKWk patients after ET for LVOS with unknown onset. Given that LKWu patients had a similar outcome 3 months after stroke, our findings indicate that ET should not be withheld from LVOS patients if the time last known well is unknown. Instead, LKWu patients may be selected for ET using the same criteria as in LKWk patients.
